# Wnt ligands from the embryonic surface ectoderm regulate ‘bimetallic strip’ optic cup morphogenesis in mouse

**DOI:** 10.1242/dev.120022

**Published:** 2015-03-01

**Authors:** April C. Carpenter, April N. Smith, Heidi Wagner, Yamit Cohen-Tayar, Sujata Rao, Valerie Wallace, Ruth Ashery-Padan, Richard A. Lang

**Affiliations:** 1 Visual Systems Group, Abrahamson Pediatric Eye Institute, Division of Pediatric Ophthalmology, Cincinnati Children's Hospital Medical Center, Cincinnati, OH 45229, USA; 2 Department of Human Molecular Genetics and Biochemistry, Sackler Faculty of Medicine andSagol School of Neuroscience, Tel-Aviv University, Ramat Aviv, Tel Aviv 6997801, Israel; 3 Vision Science Research Program, Toronto Western Research Institute, University Health Network, Toronto, Ontario M5T 2S8, Canada; 4 Department of Ophthalmology and Vision Science, University of Toronto, Toronto, Ontario M5T 2S8, Canada; 5 Division of Developmental Biology, Cincinnati Children's Hospital Medical Center, Cincinnati, OH 45229, USA; 6 Department of Ophthalmology, College of Medicine, University of Cincinnati, Cincinnati, OH 45229, USA

**Keywords:** Morphogenesis, Optic cup, Wnt ligands

## Abstract

The Wnt/β-catenin response pathway is central to many developmental processes. Here, we assessed the role of Wnt signaling in early eye development using the mouse as a model system. We showed that the surface ectoderm region that includes the lens placode expressed 12 out of 19 possible Wnt ligands. When these activities were suppressed by conditional deletion of wntless (*Le-cre; Wls^fl/fl^*) there were dramatic consequences that included a saucer-shaped optic cup, ventral coloboma, and a deficiency of periocular mesenchyme. This phenotype shared features with that produced when the Wnt/β-catenin pathway co-receptor Lrp6 is mutated or when retinoic acid (RA) signaling in the eye is compromised. Consistent with this, microarray and cell fate marker analysis identified a series of expression changes in genes known to be regulated by RA or by the Wnt/β-catenin pathway. Using pathway reporters, we showed that Wnt ligands from the surface ectoderm directly or indirectly elicit a Wnt/β-catenin response in retinal pigment epithelium (RPE) progenitors near the optic cup rim. In *Le-cre; Wls^fl/fl^* mice, the numbers of RPE cells are reduced and this can explain, using the principle of the bimetallic strip, the curvature of the optic cup. These data thus establish a novel hypothesis to explain how differential cell numbers in a bilayered epithelium can lead to shape change.

## Abstract (precis)

**Summary:** During optic cup morphogenesis, Wnt ligands expressed in the surface ectoderm control cell proliferation in the retinal pigmented epithelium, and thus influence bending of the neural retina.

## INTRODUCTION

Eye development begins with bilateral evagination of the diencephalon to form the optic vesicles (reviewed by Chow and Lang, 2001; Fuhrmann, 2010). Interaction between the optic vesicles and the closely overlying surface ectoderm is followed by thickening of the surface ectoderm to form the lens placode, and the generation of distinct neuroepithelial domains that give rise to Chx10 (Vsx2)-positive (Nguyen and Arnheiter, 2000) neural retina (NR), Mitf-positive (Nguyen and Arnheiter, 2000) retinal pigment epithelium (RPE) and the optic stalk (OS). Subsequently, the lens placode and the optic vesicle undergo a coordinated invagination to form the lens vesicle and a bilayered optic cup, respectively. The neuroepithelium at this stage has the potential to give rise to RPE or NR; however, appropriate signals result in an inner NR and an outer RPE (Reh and Pittack, 1995; Rowan and Cepko, 2004; Horsford et al., 2005). The anterior optic cup rim at the junction between the NR and the RPE (also called the hinge region) will produce ciliary margin structures (the iris and ciliary body) (Chow and Lang, 2001; Martínez-Morales et al., 2004). Neural crest-derived periocular mesenchyme (POM) lies between the optic vesicle and surface ectoderm at E8.5, is excluded at E9.5 as the lens placode forms, but ultimately contributes to many eye structures including components of the cornea (Gage et al., 2005).

The bilayered epithelial structure of the optic cup results from a fold-back movement that places the apices of the presumptive retina and RPE together. This fold-back morphogenesis is not restricted to the distal optic vesicle but also occurs ventrally. This movement folds the nascent vascular system into the developing eye and requires an epithelial fusion ventrally to form an intact cup. Recent work has suggested that regulation of RPE development via the Wnt/β-catenin pathway is a key factor in development of the optic cup (Fuhrmann, 2008; Westenskow et al., 2009; Eiraku et al., 2011). It has also been suggested that, in chick, a combination of Wnt and BMP signals specifies the RPE (Steinfeld et al., 2013) and controls positioning of the lens (Grocott et al., 2011).

A number of Wnt pathway components are expressed in the early optic cup and later restricted to the retina rim at the fold-back hinge-point (Fuhrmann et al., 2000; Liu et al., 2003, 2006, 2007; Cho and Cepko, 2006; Trimarchi et al., 2009). In addition, it has been shown using different Wnt/β-catenin reporter mice that there is activation of the canonical pathway in the developing anterior RPE, suggesting that there might be a role for Wnt signaling in RPE specification (Kreslova et al., 2007; Liu et al., 2007; Zhou et al., 2008; Westenskow et al., 2009). Consistent with this, the RPE marker *Mitf* is a direct target gene of the canonical Wnt pathway (Tachibana, 2000; Saito et al., 2003; Larue and Delmas, 2006; Schepsky et al., 2006) and it has been shown that β-catenin loss of function in the optic vesicle or RPE results in the loss of RPE markers (Fujimura et al., 2009; Westenskow et al., 2009, 2010; Hägglund et al., 2013). Alongside this analysis, it has been shown that when optic cups are grown from embryonic stem cells (ESCs) in culture, RPE development can be enhanced by supplementing the growth medium with recombinant Wnt ligands (Eiraku et al., 2011). It has further been shown by atomic force microscopy of ESC-derived optic cups that the RPE is relatively stiff compared with the NR (Eiraku et al., 2011). This has led to the proposal that, coupled with the apical constriction of the hinge-point cells at the retinal rim, RPE stiffness and retinal flexibility results in retinal invagination and the formation of a bilayered cup (Eiraku et al., 2011). It has been proposed that the neural tube may be the natural source of Wnt ligands required for the RPE Wnt/β-catenin response (Eiraku et al., 2011).

Here we investigated the role of Wnt ligands in early eye development in mouse using a conditional allele of the Wnt ligand transporter wntless (Wls) (Carpenter et al., 2010). We show that loss of Wls from the ocular surface ectoderm results in profound changes in eye development and morphogenesis. The data suggest that these changes result from the absence of Wnt/β-catenin signaling in the distal retinal rim and RPE progenitors. We suggest that a Wnt/β-catenin-driven expansion of the RPE is responsible for the generation of curvature in the optic cup. This might in part be driven by Wnt-dependent retinoic acid signaling in the optic cup.

## RESULTS

### Wnt ligands are expressed in both ocular ectoderm and mesenchyme

**Fig. 1.**
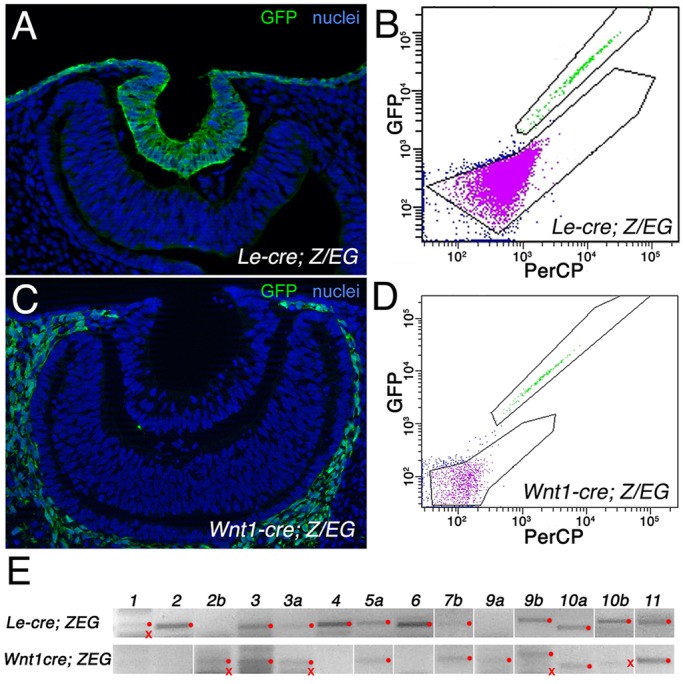
**Flow sorting and Wnt expression in ocular ectoderm and mesenchyme.** (A,C) GFP labeling in transverse cryosections of the eye region from *Le-cre; Z/EG* (A) and *Wnt1-cre; Z/EG* (C) mouse embryos at E10.5. Hoechst 33258 nuclear staining is in blue. (B,D) Flow sorting of GFP^+^ cells (green) from *Le-cre; Z/EG* (B) and *Wnt1-cre; Z/EG* (D) eye tissue at E10.5. (E) End-point PCR for labeled Wnt transcripts from flow-sorted GFP^+^ cells of the indicated genotype. Red dots indicate bands of the correct size for the particular Wnt transcript; red crosses indicate bands of incorrect size.

Wnt responses are implicated in early eye development (Smith et al., 2005; Fuhrmann, 2008; Swindell et al., 2008; Zhou et al., 2008). To define the ligand genes expressed in eye tissues that were Cre targetable, we flow sorted GFP^+^ cells from *Z/EG* mice (Novak et al., 2000) converted by *Le-cre* (Ashery-Padan et al., 2000) and *Wnt1-cre* (Brault et al., 2001); these identify, respectively, ocular ectoderm and neural crest-derived POM cells according to antibody labeling for GFP in cryosections (Fig. 1A,C). Flow sorting of GFP^+^ cells from dissected eye regions from E10.5 showed well separated populations (Fig. 1B,D). End-point RT-PCR on mRNA isolated from these GFP^+^ populations showed that many Wnt ligands were expressed. Ectoderm expressing *Le-cre* expressed the majority of the Wnt ligand family, including *Wnt1*, *2*, *3*, *3a*, *4*, *5a*, *6*, *9b*, *10a*, *10b* and *11* (Fig 1E). Sorted neural crest-derived POM cells expressed mRNA for *Wnt2b*, *3*, *3a*, *5a*, *7b*, *9a*, *9b*, *10* and *11* (Fig 1E).

### Wls is expressed broadly and can be deleted from ectoderm with *Le-cre*

One means to assess Wnt ligand activity is to delete the essential Wnt ligand transporter wntless (Wls; also known as Gpr177) (Carpenter et al., 2010; Stefater et al., 2011). Thus, to determine the consequences of the loss of Wnt activity from the surface ectoderm, including the presumptive lens, we combined a mouse *Wls* conditional allele (Carpenter et al., 2010) with the *Le-cre* driver.

**Fig. 2.**
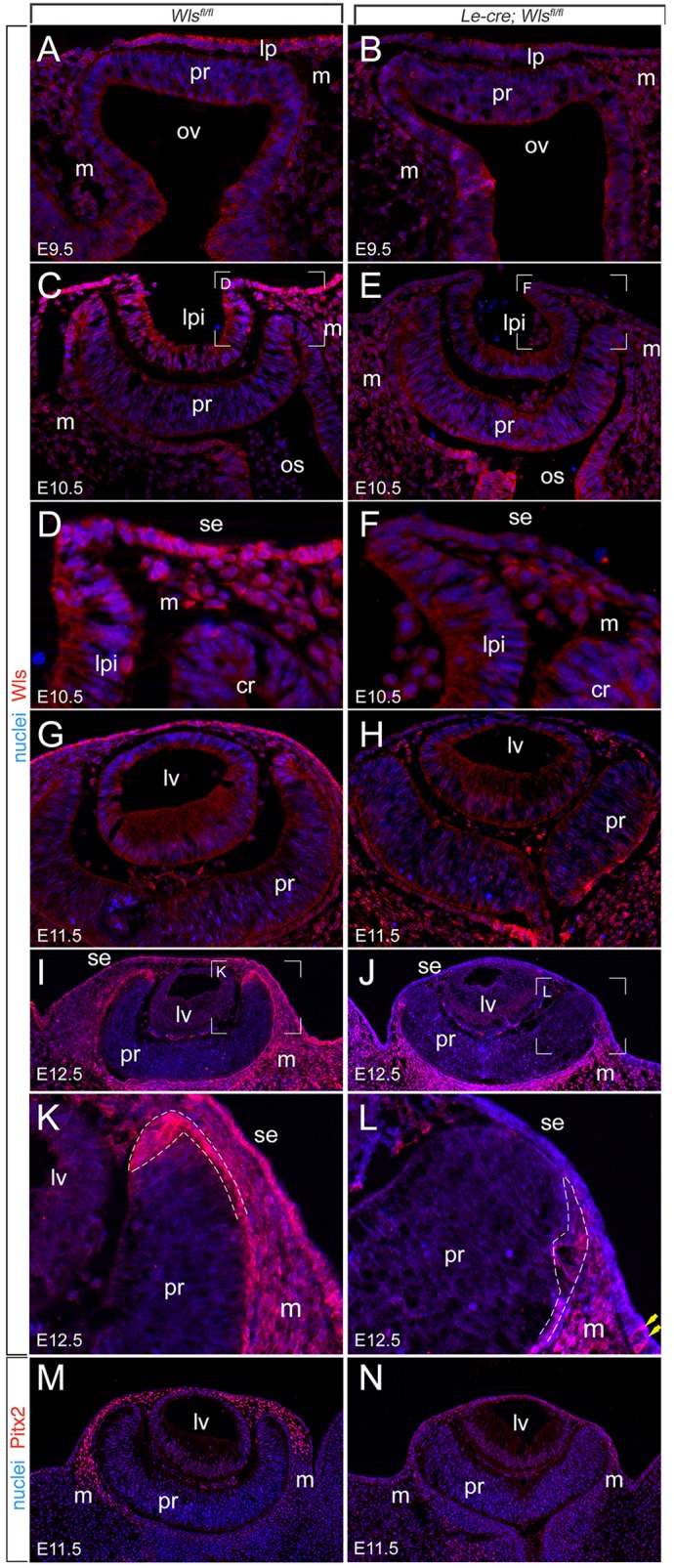
**Distribution of Wls and its deletion from *Le-cre; Wls^fl/fl^* eyes.** (A-L) Transverse cryosections of the eye region of *Wls^fl/fl^* control and *Le-cre; Wls^fl/fl^* embryos at the indicated embryonic stages, showing Wls immunofluorescence (red) and Hoechst 33258 nuclear staining (blue). The boxed regions in C,E,I,J are magnified in D,F,K,L. Dashed lines (K,L) indicate the retinal rim regions. The yellow arrows identify Wls-labeled cells at the edge of the *Le-cre* expression domain. (M,N) Immunolabeling for Pitx2 (red) and nuclear staining (blue) of E11.5 eyes of the indicated genotype. m, mesenchyme; ov, optic vesicle; pr, presumptive retina; lp, lens placode; lpi, lens pit; os, optic stalk; cr, optic cup rim; lv, lens vesicle; se, surface ectoderm.

According to immunofluorescence labeling in control mice, Wls is normally detected in the lens placode, periocular ectoderm and POM from E9.5 (Fig. 2A). At E10.5, Wls immunoreactivity in the lens pit has diminished but labeling is intense in the adjacent surface ectoderm that overlies the retinal rim (Fig. 2C,D). Wls immunoreactivity is also detected in the retinal rim and in the mesenchyme that lies between the surface ectoderm and the retinal rim (Fig. 2C,D). In *Le-cre; Wls^fl/fl^* mice, Wls immunoreactivity is lost from the lens placode and periocular ectoderm at E9.5 (Fig. 2B), confirming that the *Le-cre* driver was effective. From E10.5 and beyond in *Le-cre; Wls^fl/fl^* mice, the surface ectoderm and its derivatives remain Wls negative (Fig. 2E,F), with the exception of cells at the edge of the normal *Le-cre* expression domain (Fig. 2L, yellow arrows). Immunodetection in control mice shows that Wls is intensely expressed in presumptive corneal ectoderm at E11.5 (Fig. 2G) but that this expression is absent in the experimental mice (Fig. 2H), as would be anticipated. At E10.5 and beyond, the expression of Wls in the mesenchyme and distal retinal rim that can be observed in control mice (Fig. 2C,D,G,I,K) is diminished in *Le-cre; Wls^fl/fl^* mice (Fig. 2E,F,H,J,L). This suggests that ectoderm-derived Wnt ligands indirectly regulate the expression of Wls in the adjacent tissues and is consistent with the suggestion that *Wls* is a Wnt/β-catenin pathway target gene (Fu et al., 2009).

### Surface ectoderm Wnt ligands are crucial for eye morphogenesis

At E9.5 and E10.5, there are no obvious morphological differences between control *Wls^fl/fl^* and *Le*-*cre; Wls^fl/fl^* mice (Fig. 2A-C,E). However, at E11.5 and beyond, major differences are apparent. At E11.5 and E12.5, the loss of POM (which is Wls immunoreactive) is apparent (Fig. 2G-L), and is obvious in higher magnification images centered on the distal retinal rim (Fig. 2K,L). The loss of POM was confirmed by immunolabeling for Pitx2, a marker for POM (Fig. 2M,N) (Gage et al., 2008). The retinal rim morphology of *Le*-*cre; Wls^fl/fl^* mice is also unusual in that the cuboidal epithelial portion that resembles RPE is displaced to the outside of the eye cup. This is particularly evident at E12.5, when Wls immunoreactivity acts as a marker for the RPE-retinal transition (Fig. 2K,L, region within dashed line).

**Fig. 3.**
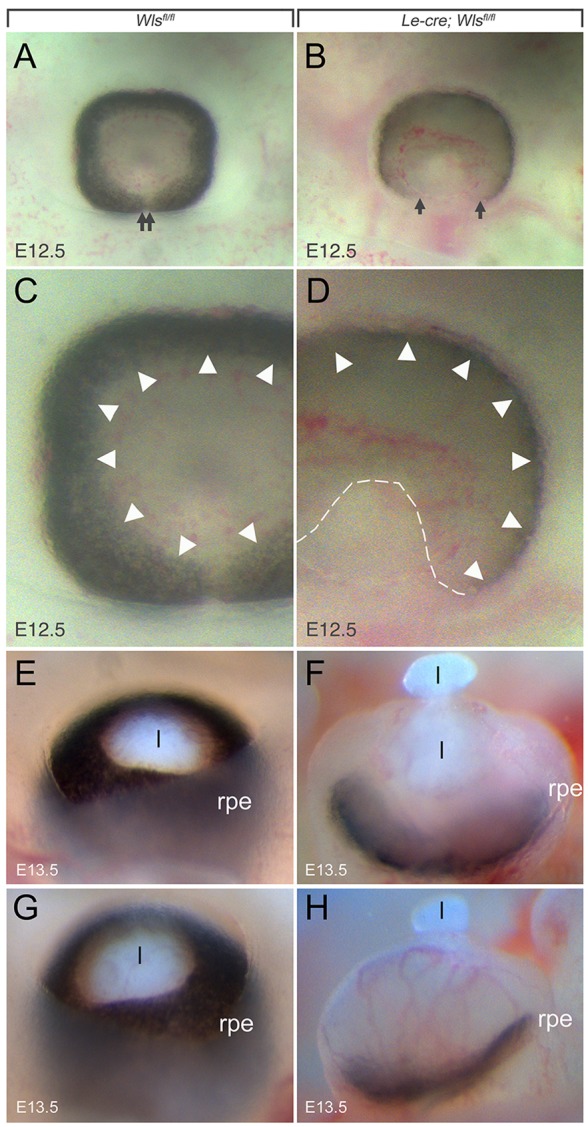
**Deleting *Wls* from presumptive lens results in a whole-eye phenotype.** Whole-mount images of E12.5 (A-D) and E13.5 (E-H) eyes of the indicated genotypes. Arrows (A,B) indicate the ventralmost aspect of the eye cup. Arrowheads (C,D) indicate the anterior edge of the pigmented epithelium. The dashed line (D) indicates the choroidal fissure that has failed to close. rpe, retinal pigmented epithelium; l, lens.

**Fig. 4.**
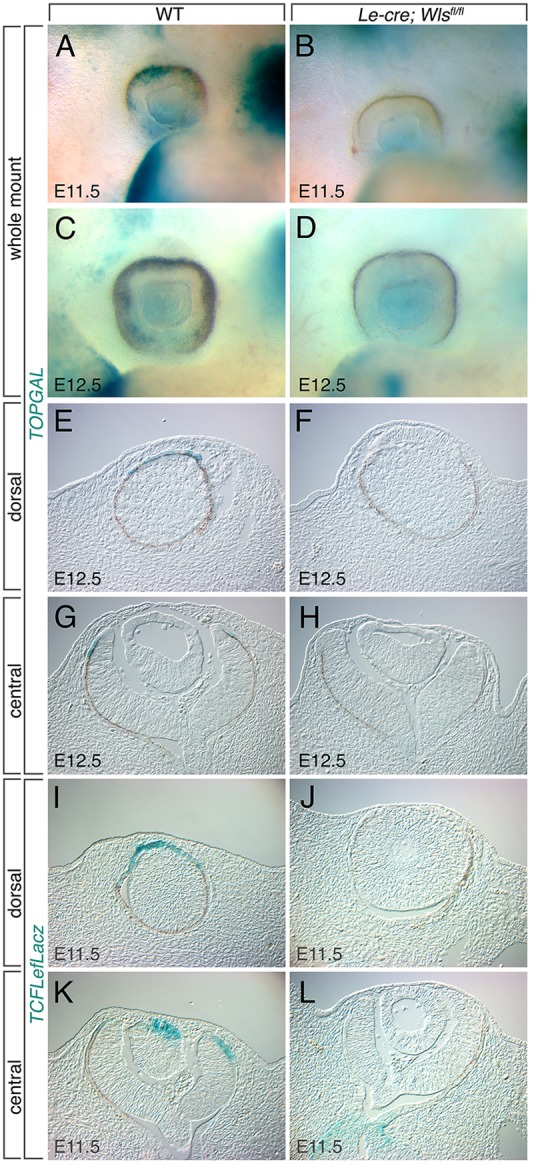
**Wnt/β-catenin reporter expression in control and in *Le-cre; Wls^fl/fl^* embryos.** (A-D) Whole-mount images of eyes from E11.5 (A,B) and E12.5 (C,D) embryos of the indicated genotypes with the *TOPGAL* reporter. WT, wild type. (E-H) Cryosections of eye regions from E12.5 embryos of the indicated genotypes with the *TOPGAL* reporter, showing dorsally (E,F) or centrally (G,H) located sections. (I-L) Cryosections of eye regions from E11.5 embryos of the indicated genotypes with the *TCF/LEFlacZ* reporter, showing dorsally (I,J) or centrally (K,L) located sections.

**Fig. 5.**
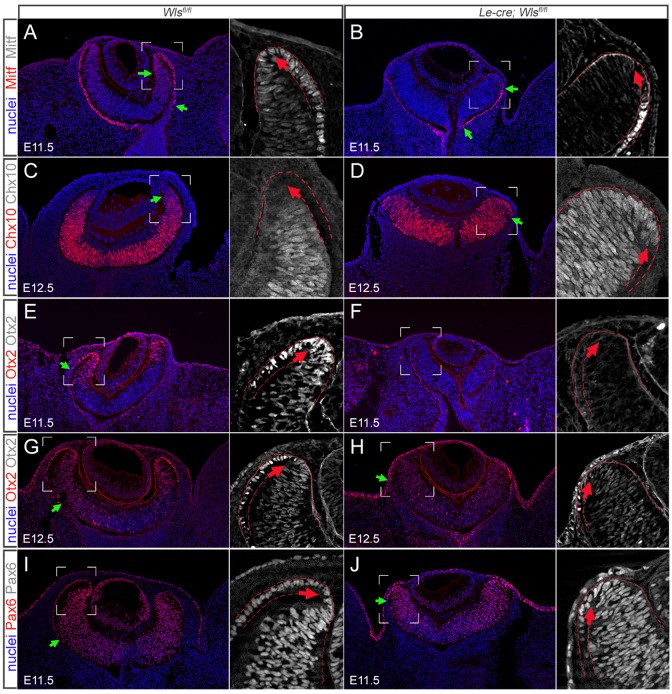
**The eye cup hinge region in *Le-cre; Wls^fl/fl^* mice.** Cryosections of the indicated embryonic stage and genotype showing immunofluorescence signal for Mitf, Chx10, Otx2 and Pax6 (red) and Hoechst 33258 nuclear staining (blue). Green arrows indicate marker transition boundaries. The boxed regions are shown to the right at higher magnification and in grayscale, with regions of interest outlined (red dashed lines and red arrows).

Whole-mount E13.5 eyes viewed axially show a ventral coloboma that reflects the failure of the ventral choroidal fissure to close (Fig. 3A-D). In addition, the distal boundary of pigmentation in the control and *Le-cre; Wls^fl/fl^* eyes differs. In section, the *Le*-*cre; Wls^fl/fl^* mice show an eye ‘cup' that is more saucer-shaped at E11.5 (Fig. 2H) and E12.5 (Fig. 2J). This change is very obvious at E13.5 when eyes are viewed laterally in whole-mount (Fig. 3E-H). In control eyes the RPE wraps around the eye cup (Fig. 3E,G), but in *Le*-*cre; Wls^fl/fl^* mice the saucer-shaped RPE does not extend distally around the eye cup (Fig. 3F,H). By E13.5, *Le*-*cre; Wls^fl/fl^* mice also show extrusion of lens material to the exterior (Fig. 3F,H). The ventral coloboma of the *Le*-*cre; Wls^fl/fl^* mice is apparent in much of this analysis (see also Fig. 4B,D, Fig. 5B,D and Fig. 7I,J) and is one feature of the phenotype similar to that of *Lrp6* mutant mice (Zhou et al., 2008). These changes in eye development indicate that Wnt ligands produced by the surface ectoderm have a crucial role in eye development and influence morphogenesis of both the mesenchyme and the eye cup.

### Surface ectoderm Wnt ligands elicit Wnt responses in the retinal rim

The retinal rim of the optic cup at the RPE transition is Wnt/β-catenin responsive (Fuhrmann et al., 2000; Liu et al., 2003, 2006, 2007; Cho and Cepko, 2006; Trimarchi et al., 2009). Thus, one explanation for the defective *Le*-*cre; Wls^fl/fl^* optic cup is that a Wnt signal from surface ectoderm normally elicits a response in the optic cup rim that helps to shape the structure. We tested this idea by assessing Wnt/β-catenin pathway reporter expression.

The *TOPGAL* (DasGupta and Fuchs, 1999) and *TCF/LEFlacZ* (Liu et al., 2003) reporter lines can, with X-gal staining, indicate the location of Wnt/β-catenin responses. In control mice, *TOPGAL* expression is observed in the eye at E11.5 in a domain that defines the boundary between the pigmented cells of the RPE and the optic cup rim (Fig. 4A). A very similar domain of *TOPGAL* activity is observed at E12.5, except that the pigmented epithelium has extended further distally (Fig. 4C). The *TOPGAL*-expressing domain is emphasized dorsally at E11.5, and has extended almost completely into the ventral half of the retinal rim by E12.5 (compare Fig. 4A and C). In *Le*-*cre; Wls^fl/fl^* mice, this domain of *TOPGAL* expression is lost at both E11.5 and E12.5 (Fig. 4B,D). The faint blue-green X-gal staining apparent behind the lens of *Le*-*cre; Wls^fl/fl^* mice is actually a domain of *TOPGAL* expression in the OS that is unchanged in the mutant (Fig. 4L).

Cryosections of E12.5 *TOPGAL* eyes subject to X-gal staining show that reporter expression is restricted to a narrow band of RPE located adjacent to the retinal rim. In dorsal coronal sections positive cells are found in a continuous stretch of pigmented epithelium (Fig. 4E), while in a more centrally located section positive cells were observed in RPE on both sides of the cup (Fig. 4G). In *Le*-*cre; Wls^fl/fl^* mice, this domain of *TOPGAL*-expressing cells is absent (Fig. 4F,H). *TCF/LEFlacZ* is an alternative Wnt/β-catenin pathway reporter (Liu et al., 2003). Although the expression patterns of *TOPGAL* and *TCF/LEFlacZ* are not identical (*TCF/LEFlacZ* is expressed in the lens epithelium at E11.5), *TCF/LEFlacZ* is, like *TOPGAL*, expressed in RPE adjacent to the retinal rim at E11.5 (Fig. 4I,K). As with the *TOPGAL* reporter, *TCF/LEFlacZ* expression is absent in *Le*-*cre; Wls^fl/fl^* mice (Fig. 4J,L). The loss of *TOPGAL* and *TCF/LEFlacZ* expression in the distal RPE of *Le*-*cre; Wls^fl/fl^* mice identifies a group of Wnt/β-catenin-responsive RPE cells adjacent to the retina-RPE transition. These data also validate the *TOPGAL* and *TCF/LEFlacZ* reporters and are consistent with the hypothesis that Wnt ligands from the surface ectoderm directly or indirectly regulate Wnt/β-catenin responses in the adjacent optic cup.

A number of target genes for the Wnt/β-catenin pathway are expressed in the retinal rim (Trimarchi et al., 2009; Westenskow et al., 2009, 2010). These include the RPE marker *Mitf* and the retinal rim marker *Otx2* (Fujimura et al., 2009; Westenskow et al., 2009, 2010). Some features of the β-catenin RPE conditional deletion, notably the ventral coloboma and unusual retinal rim morphology (Westenskow et al., 2009), are similar to those of the *Le-cre; Wls^fl/fl^* mutant. These comparisons further suggest that Wnt ligands from the surface ectoderm signal to the retinal rim. To address this further, we assessed the expression of Mitf and Otx2 in control and mutant mice. We also assessed Chx10 distribution, as it has a pattern of expression reciprocal to that of Mitf [and is repressed by Chx10 (Horsford et al., 2005)]. At E11.5, Mitf is normally found in the nuclei of presumptive RPE cells distal to the OS (Fig. 5A) but also extends around the retinal rim into the distal presumptive retina (Fig. 5A, grayscale). By contrast, in the *Le-cre; Wls^fl/fl^* mutant, Mitf does not extend around the retinal rim and is restricted to the outside of the cup (Fig. 5B and grayscale). As might be anticipated, the distribution of Chx10 shows a pattern reciprocal to that of Mitf in both the control and mutant. In the control, Chx10 is restricted to the presumptive retina at E12.5 (Fig. 5C), but in the mutant it extends around the retinal rim and marginally into the presumptive RPE (Fig. 5D).

Otx2 is normally found throughout the optic cup, but the emphasis of expression at both E11.5 (Fig. 5E) and E12.5 (Fig. 5G) is in the retinal rim. Although there is some embryo-to-embryo variation, the expression of Otx2 in the *Le-cre; Wls^fl/fl^* mutant is low overall, with the biggest contrast observed in the retinal rim (Fig. 5F,H) where expression in the control is at its highest. Finally, we also show that the distribution of Pax6 throughout the optic cup of the *Le-cre; Wls^fl/fl^* mutant is altered and does not extend as far into the RPE as in the control (Fig. 5I,J, green arrow). The changed distribution of Mitf and Otx2 in the retinal rim of the *Le-cre; Wls^fl/fl^* mutant is consistent with the hypothesis that Wnt ligands from the surface ectoderm signal to the retinal rim and normally promote development of the RPE.

### Wnt ligands from the surface ectoderm regulate proliferation in the optic cup rim and the number of cells in the RPE

Two features of the *Le-cre; Wls^fl/fl^* mutant phenotype, namely diminished RPE markers and a saucer-shaped optic ‘cup', suggested that surface ectoderm Wnt ligands might control cup morphogenesis by regulating RPE development. This supposition was based on the principle of the bimetallic strip, a temperature-sensing device in which two metals with different expansion coefficients are bonded together. When the temperature changes, the bimetallic strip bends because one side becomes longer than the other. In the same way, curvature of the biepithelial optic cup could be controlled by regulating their relative length. The flatter saucer-shaped optic ‘cup' could be a result of an oversupply of retinal cells, an undersupply of RPE cells, or a combination of both.

**Fig. 6.**
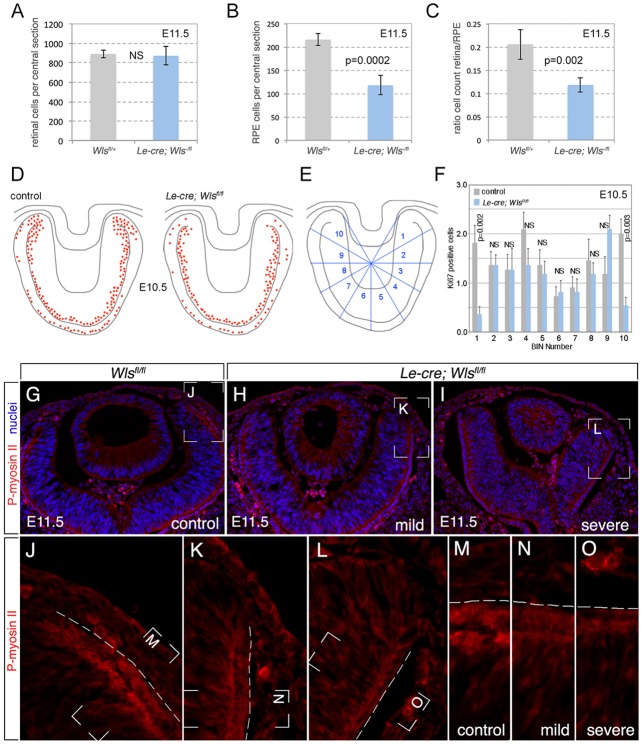
**Fewer RPE cells and reduced retinal rim proliferation in *Le-cre; Wls^fl/fl^* mice.** (A-C) Quantification of retinal cells (A), RPE cells (B) and their ratio (C) in control versus *Le-cre; Wls^–/fl^* mice. (D-F) Ki67^+^ cells are mapped onto a schematic of an E11.5 eye section for control and *Le-cre; Wls^fl/fl^* mice (D). Note the decrease in proliferation events in the retinal rim of *Le-cre; Wls^fl/fl^*. This is quantified by dividing the eye into sectors (E) and showing events in each sector for each genotype (F). Sectors 1 and 10, representing the retinal rim, show a reduction (*P*=0.002, *P*=0.003) in *Le-cre; Wls^fl/fl^*. NS, not significant. *n*=6. Error bars indicate s.e. (G-O) Assessment of phospho-myosin II immunoreactivity in control versus *Le-cre; Wls^fl/fl^* mice. (G) Control eye section. (H,I) Examples of *Le-cre; Wls^fl/fl^* mice with mild (H) and severe (I) phenotypes. The boxed regions in G-I and J-L are magnified in J-L and M-O, respectively, as two stages of magnification that emphasize the reduced phospho-myosin II immunoreactivity in the mutant RPE-retinal interface. The dashed line (J-O) marks the outer edge of the RPE.

To test this idea, we counted the number of RPE and retinal cells in E11.5 control and *Le-cre; Wls^fl/fl^* mutant mice using Mitf and Pax6 for labeling. We defined an RPE cell as one that was Mitf positive with the morphology of cuboidal epithelium, and a retinal cell as one that was Pax6 positive but without cuboidal epithelial morphology. Counts of these cell types in centrally located eye sections showed that the number of retinal cells was not significantly different between control and mutant (Fig. 6A). By contrast, the number of RPE cells in the *Le-cre; Wls^–/fl^* mutant was about half that of the control (Fig. 6B). In the control, the ratio of retinal to RPE cells was 0.21, whereas in *Le-cre; Wls^–/fl^* it was 0.12 (Fig. 6C). These data indicate that surface ectoderm Wnt ligands did not regulate the number of retinal cells, but were required for the production of cells within the RPE.

One obvious explanation for Wnt-dependent regulation of RPE cell numbers is stimulation of proliferation. To test this, we labeled E10.5 control and *Le-cre; Wls^fl/fl^* eye sections for the proliferation marker Ki67 (supplementary material Fig. S1), mapped positive cells from representative sections (*n*=11 for each genotype) onto a schematic of eye sections (Fig. 6D) and quantified Ki67^+^ cells in each of ten sectors that represented different optic cup regions (Fig. 6E,F). Visually, it was clear that rates of Ki67 labeling were much lower in the retinal rim of mutant eyes (Fig. 6D). Furthermore, quantification showed that the number of Ki67^+^ cells was significantly reduced in the two sectors (1 and 10) that represented the rim of the optic cup (Fig. 6E,F). The Ki67^+^ cells that represent the difference between the control and mutant eyes are immediately adjacent to the surface ectoderm (Fig. 6D,F). These data are consistent with the hypothesis that surface ectoderm Wnt ligands control proliferation in the optic cup rim.

In optic cups derived from ESCs (Eiraku et al., 2011) atomic force microscopy measurements show that, unlike the retina, the RPE is stiff and therefore may play a central role in shaping the optic cup. It has also been suggested that the action of contractile myosin at the retinal rim might be important for generating the RPE-retinal transition or ‘hinge' region (Eiraku et al., 2011). To determine whether the active, phosphorylated form of myosin II that is normally found in the optic cup hinge (Eiraku et al., 2011) is modulated in response to the ectodermal Wnt signals, we labeled control and *Le-cre; Wls^fl/fl^* eye cryosections with anti-phospho-myosin antibodies. The level of labeling was diminished in the mutant (Fig. 6G-O). Combined, the proliferation and phospho-myosin labeling data are consistent with a model in which surface ectoderm Wnt ligands control the number of RPE cells by promoting proliferation and may enhance stiffness in the RPE by enhancing the production of contractile actin complexes.

### Wnt signaling from ectoderm to cup is not a ligand relay via mesenchyme or RPE

Wnt ligands are lipid modified (Willert et al., 2003), insoluble and, in many cases, are thought to signal via cell-cell contact. This property of the ligands raised the possibility that when the surface ectoderm signals to the optic cup it might require the intervening mesenchyme to relay a Wnt signal. It was also possible that surface ectoderm Wnt ligands affected cup morphogenesis indirectly by eliciting local Wnt ligand production from the presumptive RPE. To test these hypotheses, we determined whether the POM was Wnt responsive and whether the *Le-cre; Wls^fl/fl^* phenotype was reproduced under conditions in which ocular mesenchyme or presumptive RPE could not make Wnt ligands.

Analysis of *TOPGAL* reporter expression in the *Le-cre; Wls^fl/fl^* mutant showed that the mesenchymal Wnt/β-catenin response was dependent on surface ectoderm Wnt ligands (supplementary material Fig. S2). However, deletion of *Wls^fl/fl^* with the mesenchymal *Wnt1-cre* (Danielian et al., 1998) produced no phenotype (supplementary material Fig. S3G-J) and no change in *TOPGAL* expression (supplementary material Fig. S3E,F) despite evidence of Wls protein loss (supplementary material Fig. S3A-D). In addition, deletion of *Wls* from RPE progenitors using *DctCre* (Zhao and Overbeek, 1999; Burke, 2008; Raviv et al., 2014) also produced no phenotypic change despite evidence of deletion (supplementary material Fig. S4). These data suggest that surface ectoderm Wnt ligands either signal to the optic cup directly or signal using a Wnt ligand-independent relay.

### Surface ectoderm Wnt ligands directly or indirectly elicit a retinoic acid signaling response in multiple eye tissues

To better understand the function of ectoderm-derived Wnt ligands, we performed a microarray analysis that compared control *Wls^fl/fl^* mice with the experimental genotype *Le-cre; Wls^fl/fl^*. RNA for the analysis was generated from E10.5 whole eye tissue containing eye cup, lens pit and POM. A comparison of transcript levels in the two genotypes revealed that 44 transcripts showed a greater than 1.5-fold change. A 2-fold reduction in the *Wls* transcript helped verify conditional deletion (Table 1). Among the remaining 43 regulated genes, a notable class comprised those (*Pitx2*, *Dkk2*, *Rbp4*, *Ttr*, *Tyr*, *Trf*, *Cryab* and *Cryge*; Table 1) that were regulated by retinoic acid (RA) or that participated in the RA pathway in some way. This, coupled with the documented association between the Wnt co-receptor *Lrp6* mutant eye phenotype and an RA signaling deficit (Zhou et al., 2008) prompted us to address the possibility that the RA signaling response was downstream of surface ectoderm Wnt ligands.

**Table 1.**
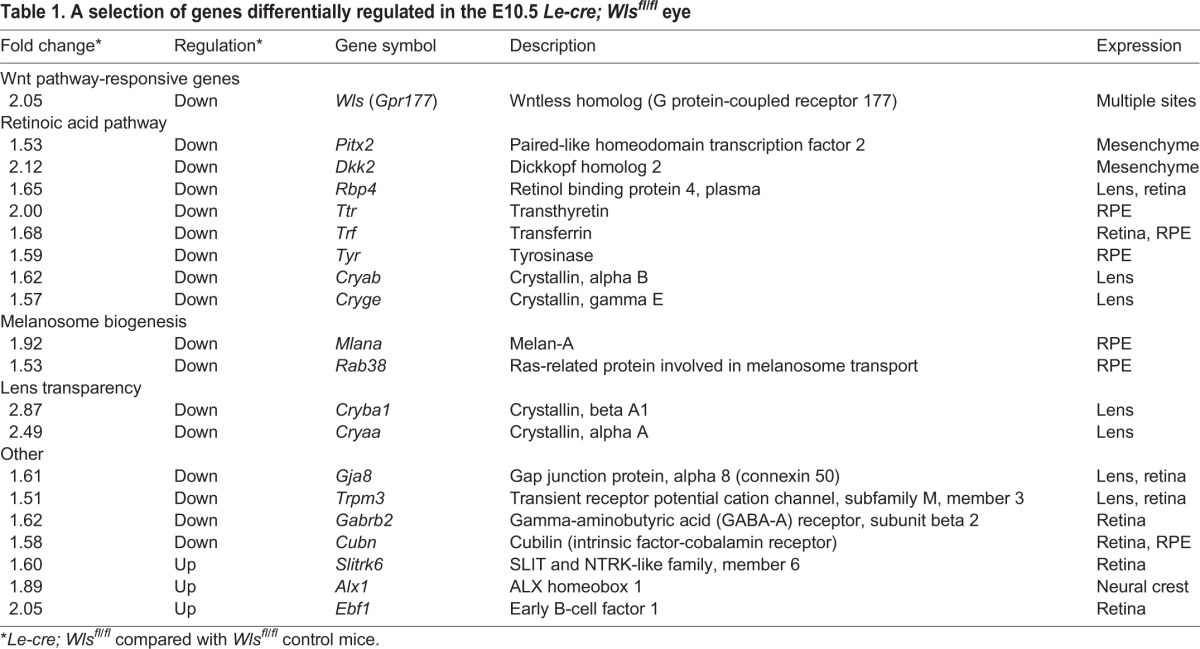
A selection of genes differentially regulated in the E10.5 *Le-cre; Wls^fl/fl^* eye

**Fig. 7.**
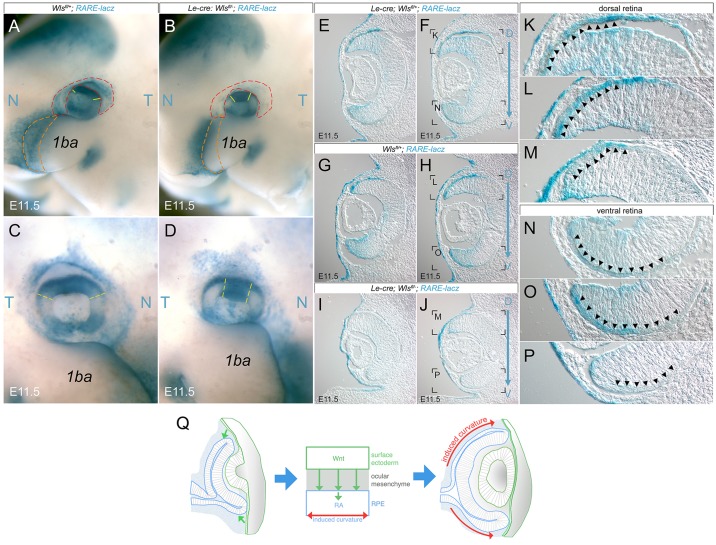
**The RA response is diminished in *Le-cre; Wls^fl/fl^* mouse eyes.** *RARE-lacZ* expression in whole-mount (A-D) and cryosections (E-P) from mice of the indicated embryonic stage and genotype. N, nasal; T, temporal; 1ba, first branchial arch. Dashed lines (A-D) indicate optic cup (red), first branchial arch rim (orange) and the dorsal domain or *RARE-lacZ* expression (yellow). Boxed regions in F,H,J are magnified in K-P as indicated. Arrowheads indicate regions of X-gal staining. (Q) Schematic describing the likely mode of Wnt-RA signaling integration required for optic cup morphogenesis. The model suggests that Wnt ligands from the surface ectoderm elicit a proliferative response in the progenitors of the RPE and that this induces curvature in the optic cup. We suggest that this response is partly dependent on the action of retinoic acid (RA).

An assessment of *Pitx2*, an RA pathway target gene, in the ocular mesenchyme of the *Le-cre; Wls^fl/fl^* mutant showed that its protein expression was greatly reduced (Fig. 2M,N), consistent with the hypothesis that RA signaling is deficient in *Le-cre; Wls^fl/fl^* mice. To assess this further, we incorporated the RA signaling reporter *RARE-lacZ* (Rossant et al., 1991) into the *Le-cre; Wls^fl/fl^* mice and determined, by X-gal staining, whether reporter activity was changed. Whole-mount images at E11.5 showed multiple domains of reduced *RARE-lacZ* activity in the ocular region. The dorsal optic cup showed a smaller domain of reporter activity (Fig. 7A-D, yellow lines). Reporter activity in the ectodermal and mesenchymal cells surrounding the optic cup was also diminished in the mutant (Fig. 7A,B, red lines). Reporter expression was absent from the first branchial arch, one region where *Le-cre* is expressed in the ectoderm (Fig. 7A,B, orange line). In some examples, changes in *RARE-lacZ* reporter expression in regions other than the dorsal optic cup were less obvious (Fig. 7C compared with D) and so we also assessed tissue sections.

In cryosections of whole-mount stained E11.5 embryos, the level of reporter activity in the *Le-cre; Wls^fl/fl^* mice was reduced in both the dorsal and ventral optic cup (Fig. 7E-J). Expression of the *RARE-lacZ* reporter in the surface ectoderm of the mutant was not obviously changed (Fig. 7E-J). At higher magnification (Fig. 7K-P), it was clear that one of the main domains of reduced X-gal staining comprises both the dorsal and ventral RPE in domains that overlap with those that express the *TOPGAL* reporter (compare Fig. 7K-P with Fig. 4 and supplementary material Fig. S2). When we examined the immunoreactivity for Raldh1 (Aldh1a1), Raldh3 (Aldh1a3) and Pax2 in dorsoventral sections of control and *Le-cre; Wls^fl/fl^* mice, we observed a very similar distribution (supplementary material Fig. S5), even though the altered shape of the retinal rim in *Le-cre; Wls^fl/fl^* mice gave the impression of higher expression ventrally. This observation is consistent with the suggestion that RA signaling does not alter dorsoventral patterning in the optic cup (Duester, 2009). These data, when coupled with prior analysis of the *Lrp6* mutant mouse (Zhou et al., 2008) and with the overlapping phenotype of *Le-cre; Wls^fl/fl^* and RA pathway mutant mice (Molotkov et al., 2006), strongly suggest that surface ectoderm Wnt ligands regulate the RA response and, in turn, optic cup morphogenesis during eye development.

## DISCUSSION

Here, we investigated the function of Wnt family ligands in eye development. We showed, by deleting the *Wls* gene in the ocular surface ectoderm at E9.5, that Wnt ligands from this source have a crucial role in eye morphogenesis. Below, we discuss in more detail the implications of these findings.

### Induced curvature in the optic cup

The eye has proven a valuable model in which to study the process of morphogenesis. Recent studies that have focused on epithelial invagination in the lens placode have shown, using conditional deletion in the presumptive lens, that the Rho family GTPases RhoA, Rac1 and Cdc42 each have a role to play and regulate epithelial cell apical constriction (Plageman et al., 2010, 2011) and cell length (Chauhan et al., 2011) as well as filopodia-dependent tethering of lens and retinal epithelium (Chauhan et al., 2009). These studies support a model in which lens pit invagination is driven by both intrinsic and extrinsic forces.

It has also been proposed that an autonomous morphogenesis program explains the acquisition of shape in the optic cup (Eiraku et al., 2011). The isolated generation of optic cups from ESCs and the measurement of the stiffness of both presumptive retina and RPE by atomic force microscopy has indicated that the RPE is rigid and the presumptive retina is flexible (Eiraku et al., 2011). Based on this observation it has been suggested that RPE stiffness is the primary determinant in shaping the optic cup. This is consistent with earlier studies showing that chick RPE could generate curvature autonomously in culture (Willbold et al., 1996). It has also been suggested that the apical constriction of cells that reside at the junction of presumptive retina and RPE (the hinge region) is important for generating the epithelial fold-back at the retinal rim and, ultimately, for invagination of the retinal component of the optic cup (Eiraku et al., 2011). Wnt signaling has been identified as a mediator of morphogenesis of ESC-derived optic cups and it has been proposed that the neural tube is the origin of these Wnt ligands (Eiraku et al., 2011).

The current study suggests some refinements to this model. First, the data indicate that at E10.5 and beyond the surface ectoderm is a crucial source of Wnt ligands that are required, directly or indirectly, for optic cup morphogenesis. This, and previous conditional *Pax6* deletion experiments (Smith et al., 2009) change the perception that optic cup development *in vivo* is entirely autonomous. Like others (Westenskow et al., 2009; Fuhrmann, 2010), we have shown that the developing RPE close to the cup rim is Wnt/β-catenin responsive. Our data showing that ectodermal *Wls* deletion results in reduced RPE cell number and a saucer-shaped optic cup are consistent with the suggestion that RPE stiffness is a driver of cup morphogenesis (Eiraku et al., 2011). In addition, we propose that the generation of RPE cells in sufficient quantity is crucial for the generation of curvature in the optic cup. This can be explained by analogy with the bimetallic strip, a temperature-sensing device that consists of two metals with dissimilar expansion coefficients that are bonded together. A change in temperature produces curvature because the two bonded strips become different lengths. In the same way, as RPE cells are produced in response to the Wnt/β-catenin–RA signaling pathway, the total length of the RPE will increase against the retinal epithelium and induce curvature of the cup. We propose that this mechanism of morphogenesis begins immediately following the coordinated invagination of presumptive lens and retina at approximately E10.5.

### Wnt/β-catenin–RA signaling linkage

Both the Wnt/β-catenin and RA signaling pathways have important roles in eye development (Fuhrmann, 2008; Cvekl and Wang, 2009; Duester, 2009). The Wnt/β-catenin pathway is active in various tissues of the eye at various stages according to activity reporters (Smith et al., 2005; Liu et al., 2006; Song et al., 2007; Fuhrmann et al., 2009). We also know that genetic modulation of Wnt/β-catenin signaling can result in defective eye development. For example, deletion of β-catenin (*Ctnnb1*) in the optic vesicle results in early eye defects including anophthalmia (Hägglund et al., 2013). Furthermore, both germline and conditional deletion of the Wnt ligand receptor *Fzd5* can result in abnormal eye development, including failure of the ventral fissure to close (coloboma) (Burns et al., 2008; Liu and Nathans, 2008). Similarly, germline mutation of the Wnt/β-catenin pathway co-receptor *Lrp6* can result in ventral coloboma (Zhou et al., 2008) or defects in the lens epithelium (Stump et al., 2003).

The RA receptor RXR and RAR families are required for eye development, as are the RA synthesis enzymes Raldh1 and Raldh3. Although there are many nuances in an interpretation of their function, there is strong evidence to indicate that RA synthesized by Raldh1 and Raldh3 in the optic cup signals to the surrounding ocular mesenchyme via Rarα-Rarβ and Rarα-Rarγ receptor heterodimers (Matt et al., 2005, 2008; Molotkov et al., 2006; Gage and Zacharias, 2009). These analyses also indicate that RA signaling is not required for patterning of dorsal and ventral retinal territories, but rather for morphogenesis of the ventral eye. Pitx2, a transcription factor required for eye development (Gage and Camper, 1997) that is normally expressed in the POM from E11.0 (Gage et al., 2008), is known to be dependent on RA signaling from the optic cup.

Prompted by similarities in the eye phenotypes of RA and Wnt pathway mutant mice, investigators have determined whether there is any regulatory linkage between the two pathways. Evidence for this linkage has been found, for example, in the *Lrp6* germline mutant mouse, in which the *RARE-lacZ* reporter suggests that RA signaling is defective (Zhou et al., 2008). Furthermore, later in eye development, there is evidence that the transcription factor Pitx2 is regulated by both RA and Wnt/β-catenin signaling (Gage and Zacharias, 2009). The current analysis strengthens the case for integration of the RA and Wnt/β-catenin signaling pathways during eye development and adds an upstream step to an optic cup morphogenesis pathway by identifying the surface ectoderm as a source of required Wnt ligands.

The simplest model of the Wnt-RA signaling pattern consistent with our data is that Wnt ligands derived from the ocular surface ectoderm signal to a region of presumptive RPE cells and in doing so enhance RA signaling (Fig. 7Q). In turn, this promotes RPE cell numbers and the establishment of curvature in the optic cup. However, this hypothesis raises the issue of how lipid-modified, insoluble Wnt signaling ligands (Willert et al., 2003) could transit the mesenchyme. It is possible that a Wnt carrier protein, perhaps a lipoprotein particle (Coudreuse and Korswagen, 2007), participates. Alternatively, specialized filopodia are known to deliver signaling ligands (Sanders et al., 2013) and it is possible that lens-to-retina filopodia (Chauhan et al., 2009) might serve this function. The absence of any phenotype resulting from *DctCre* deletion of *Wls* tends to exclude a variation of this model in which RA produced by the surface ectoderm is a diffusible signal that then elicits Wnt ligand production for Wnt/β-catenin pathway activation locally in the optic cup rim. The integration of Wnt/β-catenin and RA signaling as we describe (Fig. 7Q) is consistent with the observation that, in optic cups derived from cultured ESCs, recombinant Wnt ligands enhance RPE development and that RA is a required media supplement (Nakano et al., 2012).

Analysis performed in the chick is consistent with the current observation that surface ectoderm Wnt ligands play a role in eye development. In one analysis (Grocott et al., 2011) it was proposed that a TGFβ pathway response stimulated by POM upregulates *Wnt2b* expression in surface ectoderm and, in doing so, suppresses lens formation in non-lens ectoderm. This is consistent with the observation that in mice aberrant activation of the Wnt/β-catenin pathway in presumptive lens prevents lens development (Smith et al., 2005; Kreslova et al., 2007) and with the regulation of Wnt pathway modulators by Pax6 (Machon et al., 2010). In addition, it has been proposed that Wnt ligands from the surface ectoderm are required for specification of the RPE. Although the *Le-cre; Wls^fl/fl^* mouse only shows reduced numbers of RPE cells, not a defect in specification, this might be explained by developmental stage, as expression of *Le-cre* occurs post-induction. Thus, it might be that early in eye development ectodermal Wnts are required for RPE specification, but that later they are required to elevate the numbers of RPE cells and permit curvature in the cup.

Although the current data provide strong evidence that the surface ectoderm is a crucial source of Wnt ligands required for optic cup morphogenesis, many questions remain unanswered. The relative roles of different Wnt ligands is unclear. Our data suggest that Pax6-expressing (defined by *Le-cre*) placodal and periocular ectoderm expresses 12 out of 19 possible Wnt ligands and this raises the very simple question of what each of them does. The expressed Wnt ligands fall into both Wnt/β-catenin and non-canonical signaling ‘classes’ [but see Yu et al. (2012) for exceptions to that statement]. Of the surface ectoderm Wnt ligands, Wnt4, Wnt5a, Wnt10a, Wnt10b and Wnt11 are largely incapable of Wnt/β-catenin signaling no matter which Fzd receptor they are offered (Yu et al., 2012). By contrast, Wnt1, Wnt2, Wnt3, Wnt3a, Wnt6, Wnt7b and Wnt9b elicit Wnt/β-catenin signaling via several Fzd receptors. It will be interesting to determine whether the differential signaling activities of these ligands is a key component of shaping the eye.

## MATERIALS AND METHODS

### Transgenic mice

Animals were housed in a pathogen-free vivarium in accordance with institutional policies. Embryos were staged and harvested as described previously (Smith et al., 2005). Genetically modified mice used in this study were: *Le-Cre* (Ashery-Padan et al., 2000), *Wls^fl^* (Carpenter et al., 2010), *Z/EG* (Novak et al., 2000), *Wnt1-cre* (Danielian et al., 1998), *DctCre* (Zhao and Overbeek, 1999; Burke, 2008; Raviv et al., 2014), *TOPGAL* (DasGupta and Fuchs, 1999), *RARE-lacZ* (Jackson, cat #008477) and *TCF/LEFlacZ* (Liu et al., 2003).

### Immunofluorescence and X-gal staining

Immunofluorescence and X-gal staining were performed as described (Smith et al., 2005). C-terminal rabbit polyclonal antibody to Wls was produced with peptide sequence CDGPTEIYKLTRKEAQE (Invitrogen Custom Services), and is available at Seven Hills Bioreagents. Immunofluorescent antibody dilutions were: rabbit polyclonal Pitx2 (1:2000; a gift from Phil Gage, University of Michigan, Ann Arbor, MI, USA), rabbit polyclonal Wls (1:1000), guinea pig polyclonal Chx10 (1:1000; a gift from Sam Pfaff, Salk Institute, La Jolla, CA, USA), rabbit polyclonal Pax6 (1:2000; Covance #PRB-278P), rabbit polyclonal Mitf (1:2500; gift from Hans Arnheiter, NIH, Bethesda, MD, USA), rabbit polyclonal Raldh3 (1:8000; gift from Kenneth Campbell, Cincinnati Children's Hospital Medical Center, OH, USA), Otx2 (1:500; Millipore #AB9566), P-myosin II (1:200; Genetex #22480), Pax2 (1:100; Abcam ab38738), Raldh1 (1:500; Abgent #AP1465C) and Ki67 (1:1000; NeoMarkers Rm9106-so). Alexa Fluor secondary antibodies were obtained from Invitrogen and used at 1:1000 dilution. All sections were counterstained with Hoechst 33342 (Sigma #B-2261) for visualization of nuclei. All statistical analysis was performed using Student's *t*-test. *P*<0.05 was considered significant.

### Detection of Wnt ligand mRNAs

Developing eye regions at E10.5 were isolated from mouse litters of the following crosses: *Le-cre; Z/EG* and *Wnt1-cre; Z/EG*. GFP^+^ cells were flow sorted with a FACS Aria II running Diva software (BD Biosciences). RNA was isolated using the RNeasy Kit (Qiagen). End-point PCR was performed using primer sequences for Wnts 1-11 from the qPrimerDepot database.

### Generation of RNA and microarrays

E10.5 embryos were dissected into cold PBS. Eye regions were removed using the MC-2010 Micro-Cautery Instrument (Protech International) and flash frozen in 30 µl RLT buffer from the Qiagen Micro RNeasy Kit. Samples from two to four embryos of the same genotype were combined to create a single sample containing four to eight eye regions. Tissue was then processed for RNA isolation using a standard protocol from the Micro RNeasy Kit to produce 20 ng total RNA. Affymetrix GeneChip Mouse Genome 430 2.0 gene expression arrays were analyzed using an Agilent Bioanalyzer. Microarrays for each genotype were performed in triplicate (*N*=3) and the data were averaged during the analysis and grouping of genes. Microarray data are available in the GEO repository under accession number GSE65154.

## Supplementary Material

Supplementary Material
