# Supplementary material for: Wnt ligands from the embryonic surface ectoderm regulate ‘bimetallic strip’ optic cup morphogenesis in mouse
Source: Development. 2015 Mar 1;142(5):972–82. doi: 10.1242/dev.120022 (PMC4352985; doi:10.1242/dev.120022)
Supplement: Supplementary Material [file supp_142_5_972__index.html]

Supplementary Material 

# Wnt ligands from the embryonic surface ectoderm regulate ‘bimetallic strip’ optic cup morphogenesis in mouse

## DEV120022 Supplementary Material

**Files in this Data Supplement:**

- Supplementary Material
