# Supplementary material for: Wnt ligands from the embryonic surface ectoderm regulate ‘bimetallic strip’ optic cup morphogenesis in mouse
Source: Development. 2015 Mar 1;142(5):972–82. doi: 10.1242/dev.120022 (PMC4352985; doi:10.1242/dev.120022)
Supplement: Supplementary Material [file supp_142.5.972_DEV120022supp.pdf]

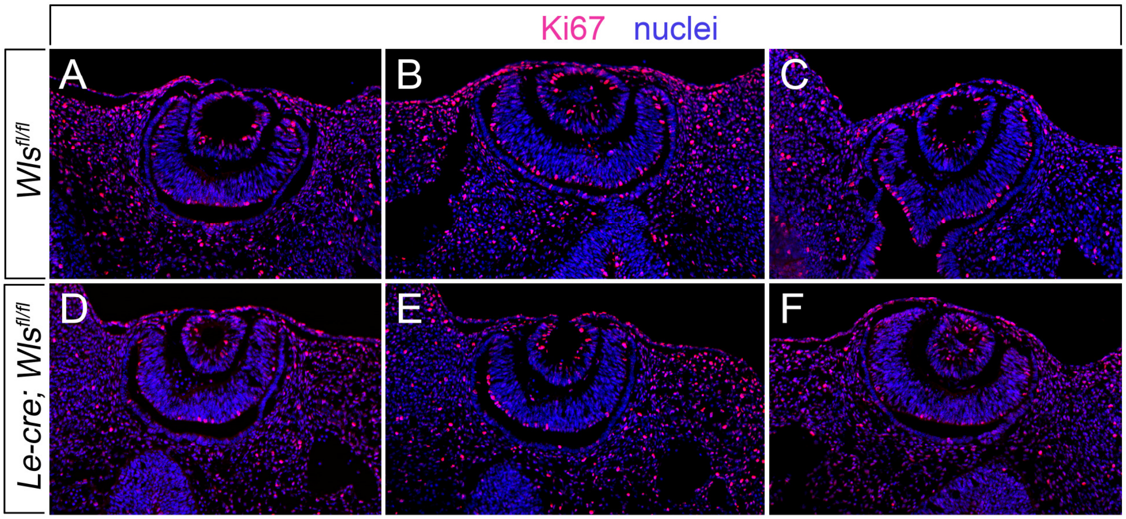

Fig. S1. Ki67 labeling in *Le-cre; Wls$^{fl/fl}$* and *Wls$^{fl/fl}$* mice

Representative examples of Ki67 labeling (red) in *Wls$^{fl/fl}$* (A-C) and *Le-cre; Wls$^{fl/fl}$* (D-F) eye region cryosections at E10.5. Nuclei are labeled blue with Hoechst 33258.

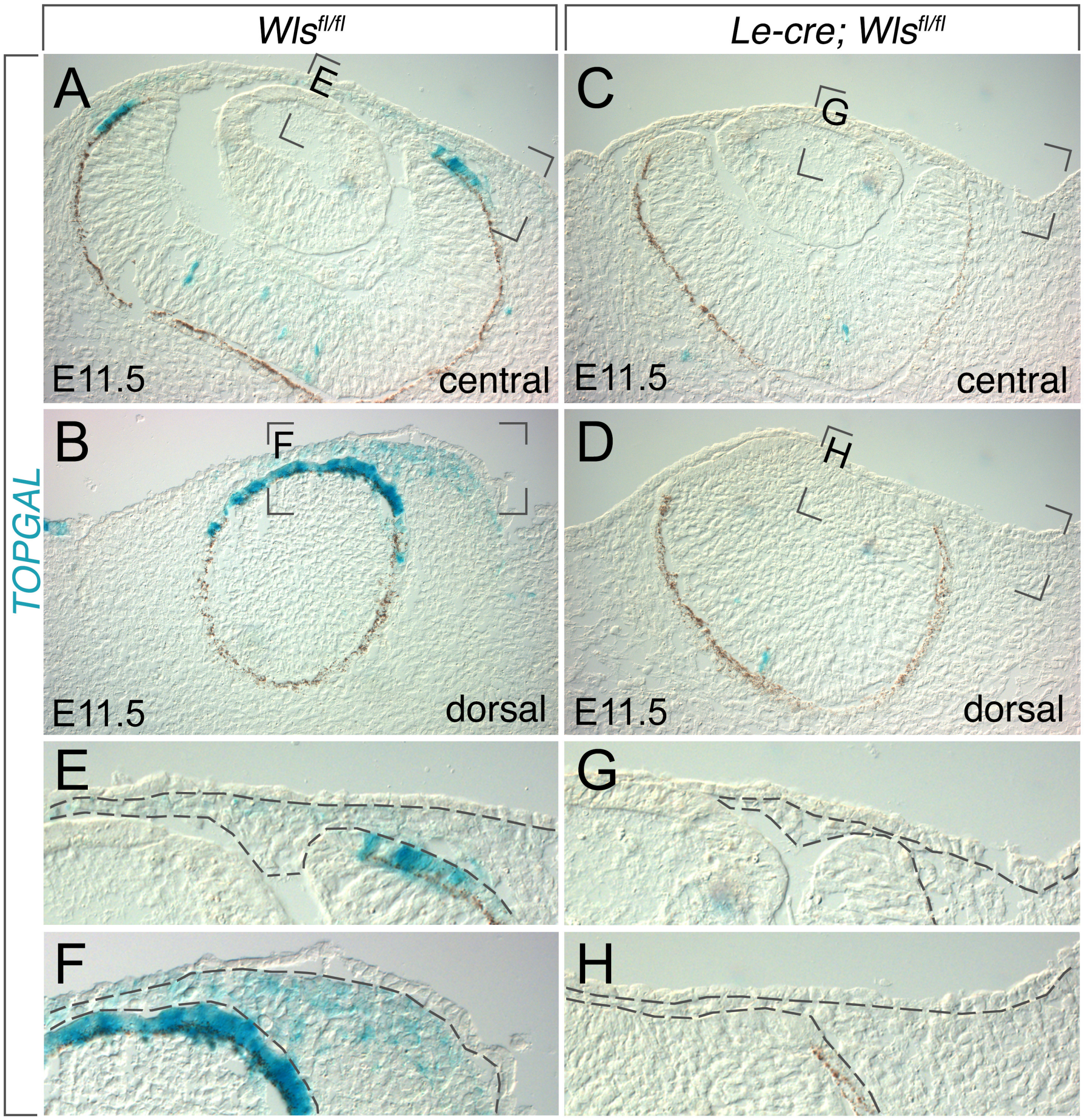

Fig. S2. *TOPGAL* expression in *Le-cre; Wls$^{fl/fl}$* and *Wls$^{fl/fl}$* mice

(A-H) Transverse sections showing X-gal labeling for *TOPGAL* in mice of the labeled genotypes and ages. Panels E-H are magnified regions of panels A-D, as shown. (A,C) Transverse sections that are centrally located and (B,D) sections that are dorsally located within the mouse eye.

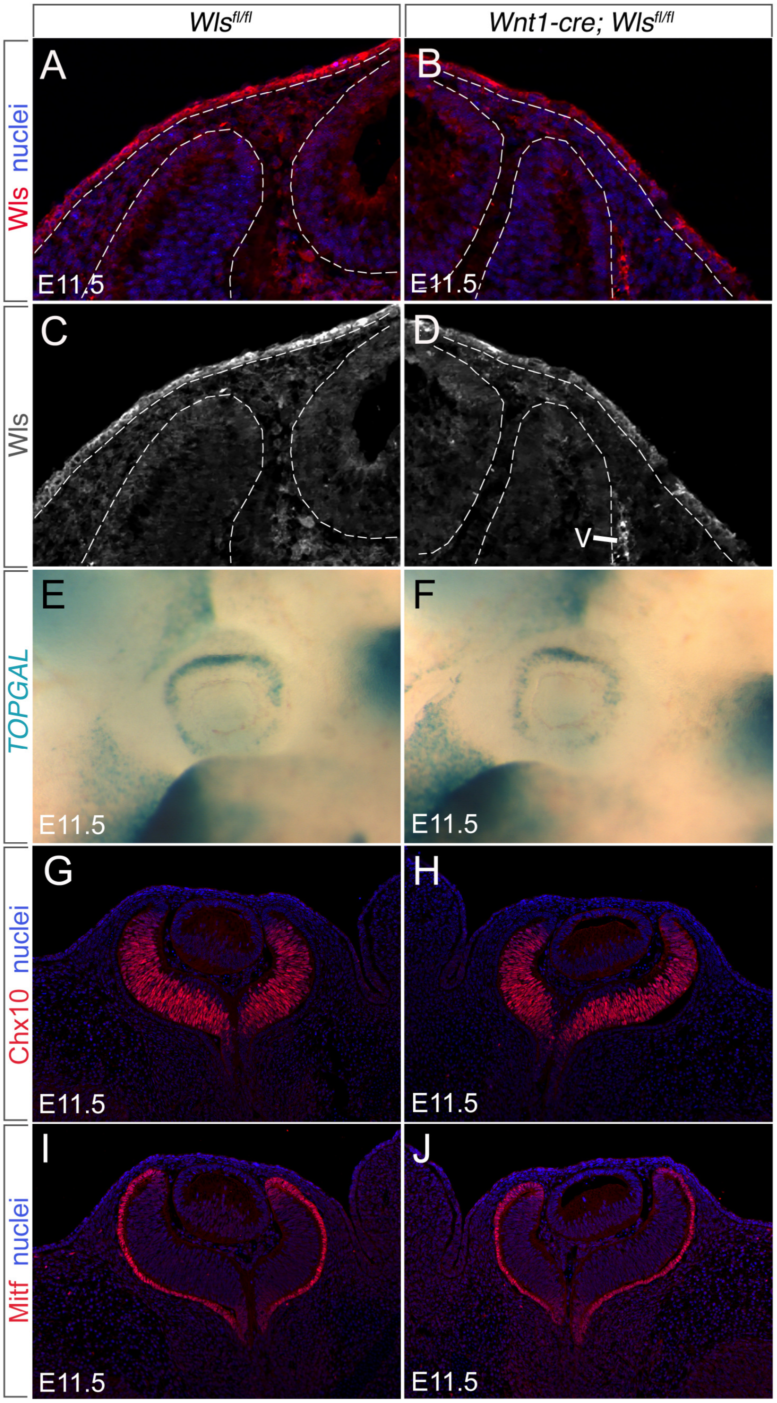

Fig. S3. Wls, *TOPGAL* and marker expression in *Wnt1-cre; Wls$^{fl/fl}$* mice

(A-D) Comparison of Wls immunoreactivity in control *Wls$^{fl/fl}$* and experimental *Wnt1-cre; Wls$^{fl/fl}$* eyes at E11.5. The region bordered by the dashed line is the ocular mesenchyme and shows reduced immunoreactivity in the mutant. This is clearest in the grayscale images (C,D). Vascular structures (V) remain strongly Wls positive. (E,F). X-gal labeling in whole-mount eyes of *Wls$^{fl/fl}$* and *Wnt1-cre; Wls$^{fl/fl}$* eyes at E11.5. (G-J) Immunofluorescence labeling for the indicated markers (red) in E11.5 cryosections from mice of the indicated genotypes. Hoechst 33258 nuclear labeling is in blue.

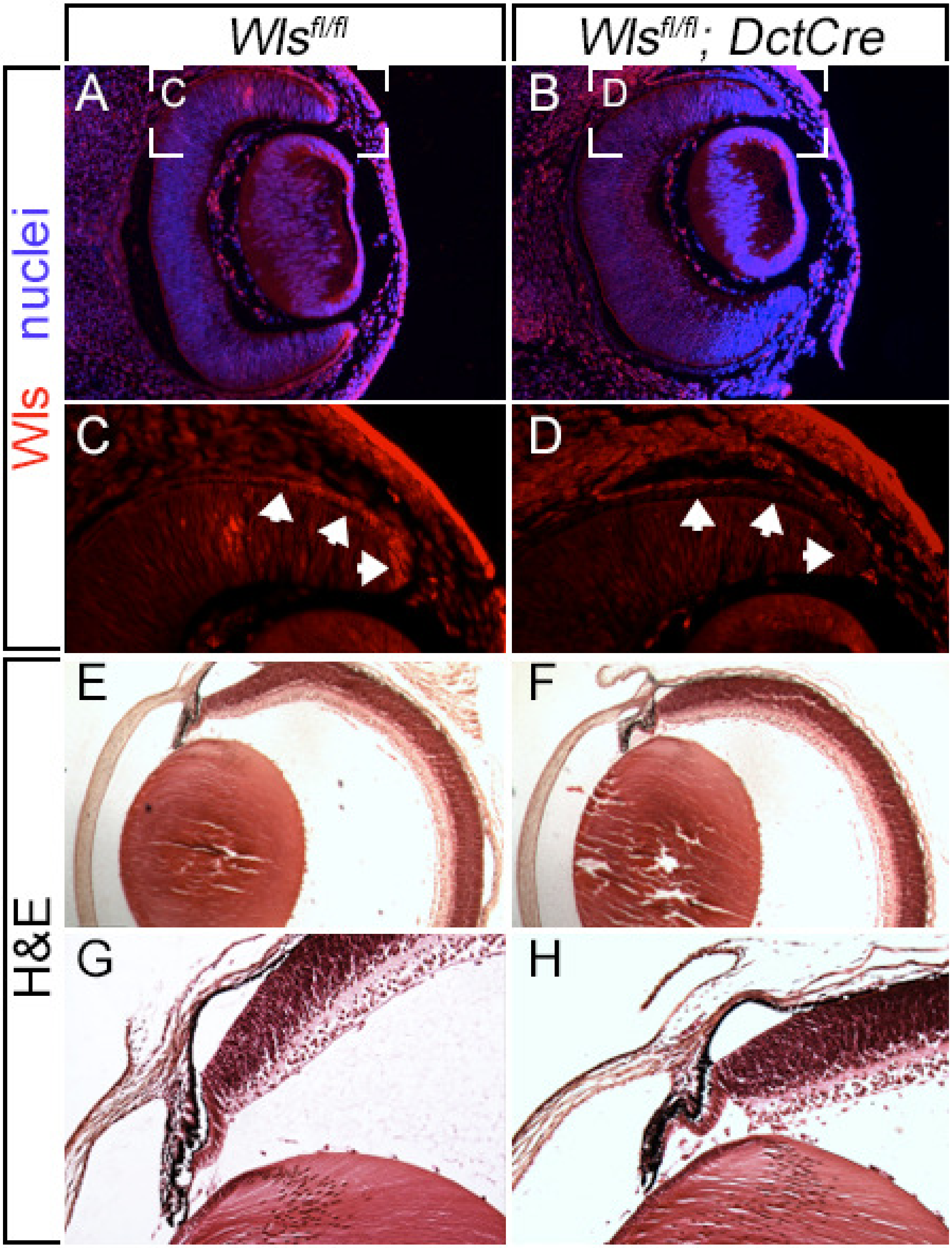

Fig. S4. *Wls* deletion with *DctCre* does not effect eye development

(A-D) Eye sections from *Wls*$^{fl/fl}$ and *Dctcre; Wls*$^{fl/fl}$ eyes at E11.5 labeled with Wls antibodies and for nuclei with Hoechst 33258. (C,D) Magnified regions to indicate the loss of Wls immunoreactivity in the distal RPE and hinge region of the optic cup (white arrows). (E-H) Compared with a control eye at P1 (E,G), the *Dctcre; Wls*$^{fl/fl}$ eye (F,H) shows normal morphology according to H&E staining.

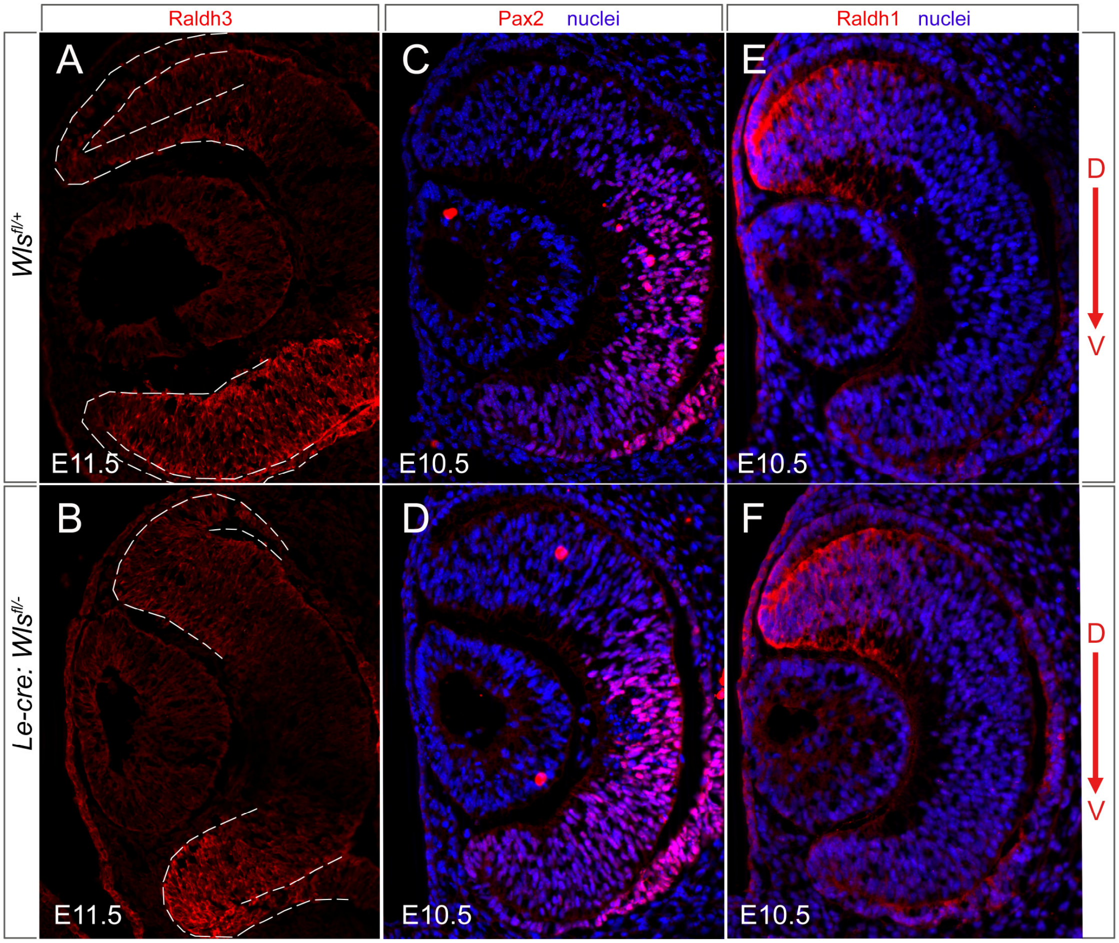

Fig. S5. Dorsal and ventral marker expression is normal in *Le-cre; Wls$^{fl/fl}$* mice

(A-F) Immunolabeling for Raldh3, Pax2 and Raldh1 (red) in *Wls$^{fl/fl}$* and *Le-cre; Wls$^{fl/fl}$* eye sections at E11.5. Nuclei are labeled in blue. D, dorsal; V, ventral. (A,B) Dashed white lines outline the optic cup rim. The D-V distribution of these markers appears normal.
